# Molecular Mechanisms Regulating Vascular Endothelial Permeability

**DOI:** 10.3390/ijms25126415

**Published:** 2024-06-11

**Authors:** Rio Wakasugi, Kenji Suzuki, Takako Kaneko-Kawano

**Affiliations:** Graduate School of Pharmacy, Ritsumeikan University, 1-1-1 Noji-higashi, Kusatsu 525-8577, Shiga, Japan; ph0180ei@ed.ritsumei.ac.jp (R.W.); kenjiszk@ph.ritsumei.ac.jp (K.S.)

**Keywords:** endothelial cell, permeability, VE-cadherin

## Abstract

Vascular endothelial cells form a monolayer in the vascular lumen and act as a selective barrier to control the permeability between blood and tissues. To maintain homeostasis, the endothelial barrier function must be strictly integrated. During acute inflammation, vascular permeability temporarily increases, allowing intravascular fluid, cells, and other components to permeate tissues. Moreover, it has been suggested that the dysregulation of endothelial cell permeability may cause several diseases, including edema, cancer, and atherosclerosis. Here, we reviewed the molecular mechanisms by which endothelial cells regulate the barrier function and physiological permeability.

## 1. Introduction

Endothelial cells form a continuous monolayer lining the inner wall of all blood vessels, and they regulate various vascular functions, such as inflammation, angiogenesis, hemostasis, and vasodilation. The endothelial cell layer is a selective barrier that restricts the extravasation of macromolecules and cells while transporting gases and nutrients into tissues [1]. This barrier is essential for maintaining homeostasis. Therefore, vascular barrier dysfunction is closely related to various pathogeneses, including edema, asthma, inflammatory airway diseases, cancer, and atherosclerosis. 

Blood exiting the heart passes through the aorta and then enters the branching arteries and arterioles, which are connected to capillaries throughout the body. The peripheral blood returns to the heart through the venules, veins, and vena cava. Endothelial cells line the interior of all vessels. Large arteries and veins form structures consisting of overlapping layers of endothelial cells, basement membranes, and smooth muscle cells [2]. Capillaries and small veins are surrounded by an endothelial cell layer, basement membrane, and pericytes [2]. Vascular permeability differs among the organs, depending on their physiological function. Accordingly, capillaries have different types of endothelia: continuous, fenestrated, or discontinuous, depending on the specific functions of the organs that they supply. In contrast, the endothelium of arteries and veins forms a continuous monolayer [2]. Fenestrated endothelia can be found in the capillaries of tissues involved in filtration and secretion, such as those of the exocrine and endocrine glands, kidney glomeruli, and intestinal mucosa; while discontinuous endothelia of the capillaries can be found in the sinusoidal vascular beds of the liver and bone marrow [2]. In the brain, the blood–brain barrier consists of endothelial cells, basement membranes, pericytes, and termini of astrocytic projections, which strictly limit the exchange of substances [3]. 

Most microvessels have limited permeability under normal conditions because the endothelium is continuous and it is surrounded by a basement membrane and some pericytes [2,4]. A strict regulation of vascular permeability is essential for maintaining organ and tissue functions. The disruption of tissue-specific vascular permeability is closely associated with various diseases, such as asthma, inflammatory airway disease, and cancer [1]. Therefore, elucidating the mechanisms controlling vascular permeability is essential to establish therapeutic strategies for such diseases. Here, we outlined the molecular mechanisms that regulate the barrier function of endothelial cells.

## 2. Endothelial Barrier Structure

**Figure 1:**
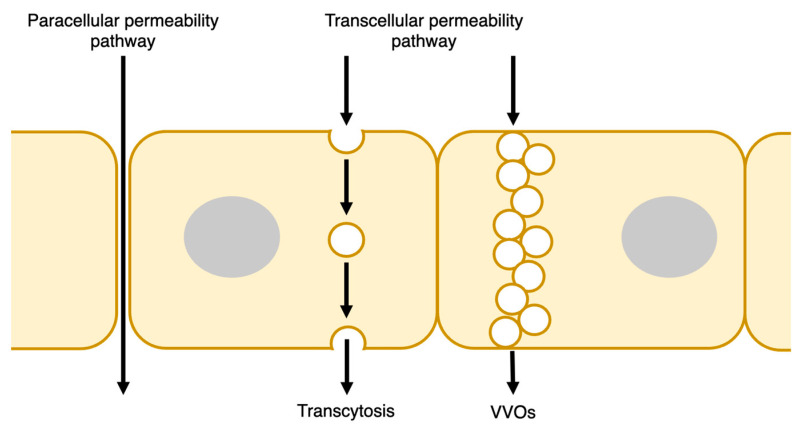
Paracellular and transcellular pathways. Endothelial cells form selective barriers in the inner lumen of vessels. Macromolecules are extravasated from the blood through paracellular and transcellular pathways. In the paracellular pathway, macromolecules pass between endothelial cells by opening the endothelial junctions. In the transcellular pathway, molecules are transported across endothelial cells through vesicle-based transcellular pathways and vesiculovacuolar organelles (VVOs).

The endothelial barrier regulates the exchange of fluids and solutes—including plasma proteins and cells, particularly leukocytes—between blood and tissues. Under quiescent conditions, the endothelial barrier suppresses the permeability of macromolecules and cells. When inflammation occurs, various mediators act on endothelial cells to increase their permeability, causing the extravasation of large molecules and cells such as plasma proteins and leukocytes. Two routes, the transcellular and paracellular pathways, regulate the leakage of substances into the extravasation space [5] (Figure 1). Leukocytes also extravasate through transcellular and paracellular routes during inflammation. Other excellent reviews have discussed this process in detail [6]. In this study, we focused on the molecular permeability mechanisms of the endothelium.

### 2.1. Transcellular Pathway

Fluids and solutes are transported across the endothelial cytoplasm from the vessel lumen to the extravascular space —at the basement membrane side—via the transcellular route (Figure 1). The transport of solutes, particularly those of macromolecules, involves a vesicle-based transcellular transport pathway, which is also known as transcytosis. Transcytosis involves endocytosis and exocytosis on opposite plasma membranes [7]. In this pathway, caveolin is an essential molecule that regulates the endothelial barrier function, and albumin and low-density lipoprotein (LDL) interact with gp60 and scavenger receptor class B type 1 (SR-B1), respectively, to induce caveolin-coated internalization [7,8,9]. Dynamin, a GTPase, accumulates in the caveolar neck region and induces the constriction (pinch-off) of caveolae buds from the luminal plasma membrane into endothelial cells [10]. Caveolae internalization depends on tyrosine phosphorylation of caveolin-1 and dynamin-2 via Src kinase [11]. Internalized caveolin-coated vesicles fuse to the plasma membrane of the extravasation side. During exocytosis, SNARE proteins mediate the formation of vesicles that doke and fuse to the plasma membrane, allowing caveolin vesicles to release their contents [12]. 

Vesiculovesicular organelles (VVOs) have also been reported as organelles with the endothelial transcellular permeability of macromolecules. VVOs are composed of numerous continuous uncoated vesicles or vacuoles that enable macromolecules to cross the cytoplasm from the luminal to the abluminal side. VVOs have been found in the tumor microvasculature, venules associated with allergic inflammation, and the endothelium of normal venules [13]. Vascular endothelial growth factor (VEGF), serotonin, and histamine induce macromolecular extravasation through VVOs [13]. 

### 2.2. Paracellular Pathway

**Figure 2:**
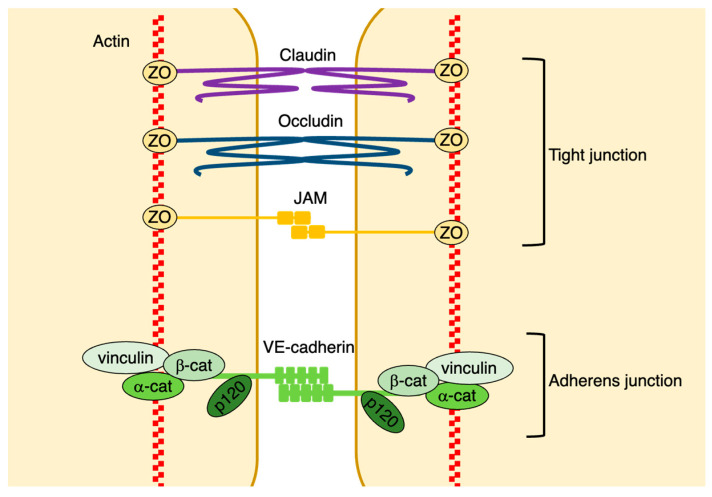
Structure of endothelial cell–cell junctions. Endothelial cell–cell junctions mainly consist of adherens, tight, and gap junctions. VE-cadherin is a component of adherens junctions, and homophilic interactions occur between the extracellular domains of these VE-cadherins. Additionally, the intracellular domain of VE-cadherins interacts with the actin cytoskeleton through adaptor proteins. Claudins and occludins are the major components of tight junctions. Their extracellular domains form tight junctions between endothelial cells, whereas their intracellular domains bind to the actin cytoskeleton via zona occludens (ZO) proteins.

The paracellular route is controlled by the opening and closing of endothelial cell adhesions. Two major junctional complexes, tight and adherent junctions, play essential roles in controlling endothelial paracellular permeability. Gap junctions are also involved in controlling endothelial permeability (Figure 2).

#### 2.2.1. Adherens Junctions

Adherens junctions are mainly comprised of vascular endothelial cadherin (VE-cadherin); therefore, this molecule plays a critical role in the control of such junctions. VE-cadherin is a cadherin family protein that is primarily expressed in vascular endothelial cells. The extracellular domains of the VE-cadherins that are expressed in neighboring endothelial cells form Ca^2+^-dependent homophilic interactions [14]. In addition, p120-catenin binds directly to the membrane peripheral region of the intracellular domains of VE-cadherins, suppressing their internalization and stabilizing them on the plasma membrane [15,16]. It has also been shown that β-catenin and γ-catenin (plakoglobin) bind to the VE-cadherin COOH-terminal intracellular domain. This complex interacts with α-catenin, which is associated with the actin cytoskeleton [6]. The VE-cadherin/catenin complex interacts with several other proteins, including actin-binding proteins such as vinculin. The interaction between the VE-cadherin/catenin complex and the actin cytoskeleton is essential for stabilizing adherens junctions [17] (Figure 2). Under steady-state conditions, VE-cadherin is associated with the cortical actin bundle, forming continuous VE-cadherin adhesions [17]. In contrast, endothelial permeability upregulation leads to the formation of gaps between endothelial cells through the alteration of actin formation and the distribution of VE-cadherin [17]. Moreover, vascular permeability-promoting factors induce actin cytoskeleton remodeling. During this process, radial contractile actin bundles connect to discontinuous cell–cell junctions, and actomyosin contraction promotes gap formation between endothelial cells, disrupting continuous endothelial adhesion and increasing endothelial permeability [18]. Under these conditions, even though gaps are formed at cell–cell contact sites, cell adhesion is not entirely disrupted, because adherens junctions maintain the connection between endothelial cells via the tips of filopodia-like protrusions [1]. The tip region of the filopodia maintains homophilic connections of tight and adherens junctions. In contrast, in areas that lose junctions and form gaps, VE-cadherin is internalized, recycled, or degraded through ubiquitination [19]. However, the mechanisms triggering VE-cadherin internalization remain unclear.

**Figure 3:**
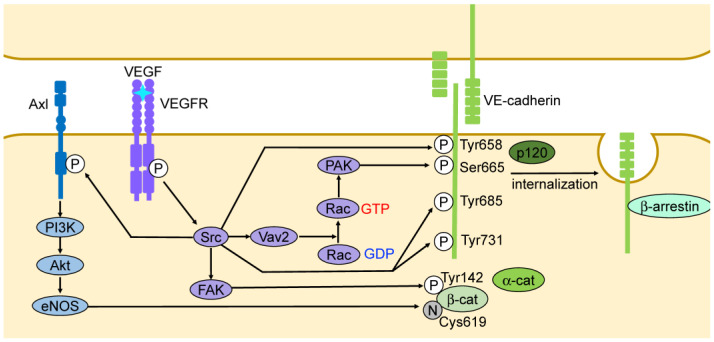
Phosphorylation of VE-cadherin and VEGF regulate adherens junctions. VE-cadherin phosphorylation controls endothelial cell adhesion stabilization. VEGF activates Src and disrupts the adherens junctions through VE-cadherin phosphorylation at Tyr658 and Tyr685. Src also activates Vav2 and FAK. Vav2 activates Rac, resulting in PAK activation. PAK phosphorylates VE-cadherin at Ser665 and promotes its dissociation from p120-catenin (p120). VE-cadherin phosphorylation at Ser665 recruits β-arrestin and enhances its internalization. FAK phosphorylates β-catenin (β-cat) at Tyr142. Src also activates Axl, which induces eNOS through PI3 kinase (PI3K) and Akt pathways, leading to the nitrosylation of β-cat at Cys619.

Some vascular permeability factors and blood flow regulate the stabilization of cell adhesion through the phosphorylation of VE-cadherin tyrosine and serine residues (Figure 3). Well-known regulatory VE-cadherin phosphorylation sites include tyrosine residues 658 (Tyr658), 685 (Tyr685), and 731 (Tyr731), and serine 665 (Ser665) [20,21]. Notably, some permeability-promoting factors enhance VE-cadherin phosphorylation at Tyr658 and Tyr731 through Src; these modifications prevent VE-cadherin from interacting with p120-catenin and β-catenin, respectively [20]. Src inhibition reduces Tyr658 and Tyr685 phosphorylation of VE-cadherin and endothelial permeability. Non-phosphorylated VE-cadherin mutations at Tyr658 (Y658F) and Tyr685 (Y685F) prevent bradykinin-induced internalization, ubiquitination, and permeability [19]. Src phosphorylates VE-cadherin at these sites, thereby regulating its internalization (Figure 3). The Src-dependent phosphorylation of Vav2—a guanine nucleotide exchange factor (GEF)—leads to the activation of Rac and p21-activated kinase (PAK), which induces VE-cadherin phosphorylation at Ser665, resulting in the recruitment of β-arrestin [21] (Figure 3). The phosphorylation of VE-cadherin at Ser665 also promotes the opening of endothelial cell–cell adhesions through the internalization of VE-cadherin [21]. Focal adhesion kinase (FAK) also regulates vascular permeability through the phosphorylation of β-catenin at Tyr142, leading to the dissociation of β-catenin from VE-cadherin and disruption of adherens junctions [22].

#### 2.2.2. Tight Junctions

In endothelial cells, tight junctions consist of claudins, occludins, junction-associated molecules (JAMs), and other adhesion molecules [23]. The intracellular domains of tight junction proteins interact with scaffolding proteins, such as zona occludens (ZO), cingulin, and paracingulin [23]. These scaffolding proteins mediate the association between tight junction molecules and the actin cytoskeleton [23] (Figure 2). Tight junction molecules form characteristic strand structures that tightly connect adjacent endothelial cells [23]. However, the permeability of the endothelial barrier is affected by the composition of the tight junction complex and the number of tight junction strands [24]. Tight junction strands are more abundant in the endothelial cells of small arteries than in those of small veins [24]. In particular, tight junctions are more abundant in the endothelial cells of brain vessels and play a critical role in controlling the blood–brain barrier function [25]. There are few tight junctions in the endothelium of capillaries and post-capillary venules, where plasma protein and circulating leukocyte extravasation easily occur. Therefore, in microvascular endothelial cells, adherens junctions are the primary regulators of endothelial permeability [26]. 

Tight and adherens junctions mutually regulate each other. VE-cadherin upregulated claudin-5 expression, indicating that adherens junctions accelerate claudin-5 expression and stabilize tight junctions [27]. In contrast, ZO-1 or claudin-5 depletion induces the disruption of tight junctions, redistribution of stress fibers, and reduction of tension in adherens junction complexes [28]. Thus, tight junctions may control the stabilization of adherens junctions through ZO-1, which regulates the interaction between VE-cadherin and the cytoskeleton.

#### 2.2.3. Gap Junctions 

Gap junctions comprise hexamers of transmembrane proteins, connexins. Connexins form channels through which small molecules, such as ions and water, can pass between adjacent endothelial cells [29]. Connexin family proteins comprise 21 members. Connexin 37, 40, and 43 are expressed in endothelial cells [29]. Connexin 43 has been reported to be inversely correlated with VE-cadherin expression in lung microvessels after endotoxin-induced vascular leakage [30]. It has also been reported that connexin 40 contributes critically to lung vascular barrier failure through myosin light chain (MLC) phosphorylation by Rho-kinase in a mouse model of acute lung injury (ALI) [31].

## 3. Regulators of Vascular Endothelial Permeability

Physiological and pathophysiological conditions can open endothelial adhesion. Endothelial barrier function is regulated by various regulatory factors of endothelial permeability and fluid shear stress due to blood flow. Importantly, VE-cadherin phosphorylation and small GTPases regulate the stabilization of endothelial adhesions through endocytosis and actomyosin contraction.

### 3.1. Small GTPases

The Rho family of small GTPases regulates actin stress fibers and cortical actin filaments. Actin stress fibers pull junctions and increase endothelial permeability, whereas cortical actin stabilizes these junctions. Thus, Rho family GTPases strongly affect actin dynamics at endothelial adhesion junctions and regulate endothelial barriers [32].

**Figure 4:**
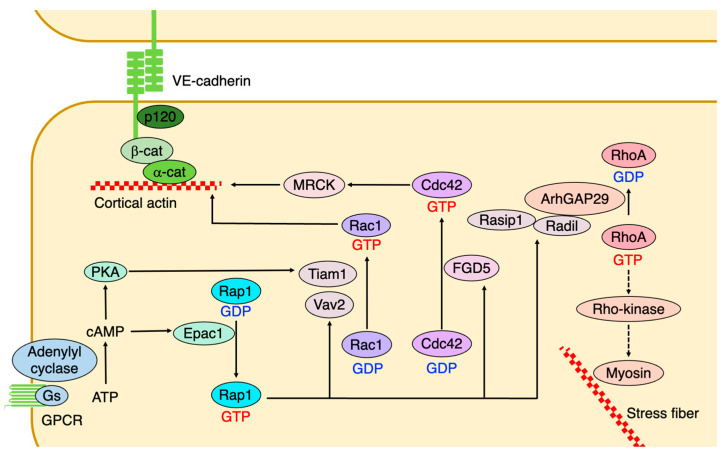
Small GTPases regulate endothelial permeability. cAMP activates PKA and Epac1—a Rap1 GEF that activates Rap1. PKA and Rap1 promote Rac1 activity through the Rac1 GEFs, Tiam1, and Vav2. Activated Rac1 enhances cortical actin and strengthens the endothelial barrier. Rap1 also activates Cdc42 via FGD5, which is a Cdc42 GEF. Activated Cdc42 induces cortical actin formation through MRCK. Meanwhile, Rap1 recruits ArhGAP29, a Rho GAP, to the junctional region and suppresses endothelial permeability by inhibiting Rho/Rho-kinase activity and stress fiber formation.

RhoA activation enhances endothelial cell permeability through Rho kinase, an effector molecule of Rho, promoting stress fiber formation and actomyosin contraction via the MLC [33,34,35] (Figure 4). Rac1 promotes the maintenance and stabilization of the endothelial barrier by promoting the assembly of cell–cell adhesion complexes and formation of cortical actin bundles [32,36,37]. However, Rac1 disrupts endothelial cell barrier function [21,38,39,40]. Thus, Rac1 exhibits opposite effects on endothelial barrier function, depending on the upstream stimuli and effectors. 

Rap1 stabilizes VE-cadherin-dependent endothelial junctions by promoting cortical actin formation via Epac1—a Rap1 GEF that is activated by cAMP [41,42,43,44]. cAMP also activates Rac1 through the Rac1 GEFs, Vav2, and Tiam1, which are activated by Epac1/Rap1 signaling and active PKA, respectively [45,46,47] (Figure 4). Furthermore, Rap1 enhances endothelial barrier function by suppressing radial actin stress fiber formation through the inhibition of RhoA/Rho-kinase activities by recruiting ArhGAP29—a GTPase-activating protein (GAP) against Rho—to junctions via Ras-interacting protein 1 (Rasip1) and Radil—effector proteins of Rap1—[48,49,50] (Figure 4). Rap1 also stabilizes adherens junctions and enhances cortical actin by recruiting FGD5—a Cdc42 GEF—at cell–cell junctions and inducing the activation of Cdc42 and its effector myotonic dystrophy kinase-related Cdc42-binding kinase (MRCK) [48] (Figure 4). 

### 3.2. Vascular Endothelial Growth Factor (VEGF) 

Increased vascular permeability by VEGF, a vascular permeability factor, has been shown to play a crucial pathophysiological role in tumorigenesis, diabetic retinopathy, and ischemia/reperfusion injury [51]. Mammalian VEGF family members include VEGFA, VEGFB, VEGFC, VEGFD, and placental growth factor (PlGF), which act through the transmembrane tyrosine kinase receptors VEGFR1, VEGFR2, and VEGFR3 [52]. VEGFR2 is the most abundant receptor protein in endothelial cells and is vital for the enhancement of vascular permeability by VEGFA [52]. VEGFR3 suppresses VEGFR2 expression and limits vascular permeability [53].

VEGF enhances the tyrosine phosphorylation of VE-cadherin via the tyrosine kinases Src and Yes [54,55,56] (Figure 3). VEGF-induced Src activation leads to Ser665 phosphorylation of VE-cadherin via PAK, recruiting β-arrestin and promoting the clathrin-dependent endocytosis of VE-cadherin [21] (Figure 3). VEGFA also promotes the binding of FAK to the cytoplasmic domain of VE-cadherin, which increases endothelial permeability through the phosphorylation of VE-cadherin at Tyr658 [57]. Additionally, VEGF promotes the binding of FAK to the cytoplasmic domain of VE-cadherin through Src, which phosphorylates β-catenin at Tyr142, dissociates β-catenin from VE-cadherin, and promotes the disruption of adherens junctions [22] (Figure 3). VEGFA has several splice variants that interact differently with basement membrane proteins and the VEGF coreceptors neuropilins [52]. Neuropilins contribute to VEGFA-induced leakage [58]. Neurophilin-1 interacts with VE-cadherin, promoting its internalization and accelerating vascular endothelial permeability initiated by histamine both in vitro and in vivo [59]. Furthermore, VEGF promotes vascular endothelial permeability by enhancing occludin transport from the cell membrane to the endosomes via phosphorylation and ubiquitination [60]. Vascular endothelial protein tyrosine phosphatase (VE-PTP) associates with VE-cadherin, strengthening its adhesion [61,62]. VEGF and other mediators that promote permeability induce the dissociation of VE-PTP from VE-cadherin, further increasing vascular permeability [63]. 

It is known that nitric oxide (NO) diffuses from endothelial cells into the surrounding vascular smooth muscle cells, causing vasodilation, increased flow, and an increased extravasation of fluids and small molecules. Notably, the suppression of endothelial NO synthase (eNOS) expression reduces VEGFA-induced vascular leakage [64]. Additionally, NO induces S-nitrosylation of β-catenin at Cys619, which results in the dissociation of β-catenin from VE-cadherin and the destabilization of adherens junctions [65] (Figure 3). 

VEGFA induces the autophosphorylation of VEGFR2 at several tyrosine residues including Tyr949 (in humans, Tyr951) and Tyr1173 (in humans, Tyr1175) [66]. The phosphorylation of Tyr949 of VEGFR2 induces an interaction with T cell-specific adaptor protein (TSAd) and activates Src, which attenuates vascular endothelial cell adhesion via the phosphorylation of VE-cadherin [67,68,69]. Mice expressing a VEGFR2 mutant that is not phosphorylated at Tyr949 exhibit no increase in vascular permeability in response to VEGF, indicating the physiological importance of Tyr949 phosphorylation in VEGFR2 [68]. Mice expressing a VEGFR2 mutant non-phosphorylated at Tyr1173 or lacking PLCγ suppressed vascular leakage [70]. These results suggest that the phosphorylation of Tyr1173 in VEGFR2 also increases vascular permeability through PLCγ.

VEGFA initiates angiogenesis by attenuating endothelial adhesion and activating cell migration, which induces the formation of tip and stalk cells [71,72]. During angiogenesis, VEGFA attenuates endothelial adhesion via a signaling pathway that induces endothelial permeability. Downstream of VEGFA, activated VEGFR2 induces Src activation through TsAd, which phosphorylates VE-cadherin, resulting in an attenuation of adhesion during sprouting [73]. VEGFR2 also promotes actin-dependent lamellipodia formation and reorganizes intercellular adhesions [74].

### 3.3. Angiopoietin

Several angiopoietins, such as angiopoietin-1, angiopoietin-2, and angiopoietin-4 (the ortholog of mouse angiopoietin-3), have been reported in humans along with the tyrosine kinase receptors Tie1 and Tie2 (TEK) [75]. Angiopoietin 1–4 are all ligands for Tie2, whereas Tie1 is an orphan receptor that interacts with Tie2 and is activated by angiopoietins through Tie2 [76]. 

**Figure 5:**
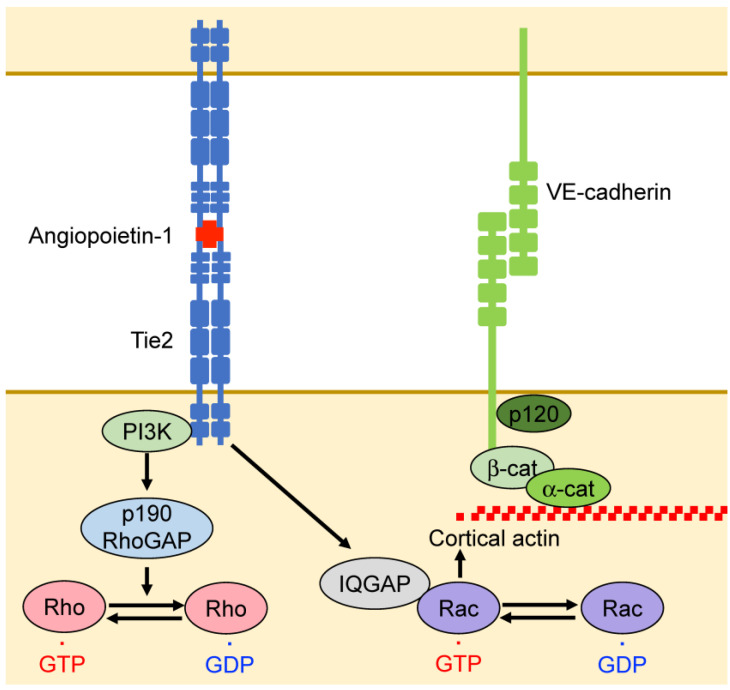
Angiopoietin regulates endothelial barrier function. Angiopoietin-1 interacts with Tie2 and controls vascular permeability by regulating the activities of RhoA and Rac1. Tie2 suppresses RhoA activation via PI3K and p190RhoGAP. Tie2 also activates Rac1 through IQ domain GTPase-activating protein 1 (IQGAP1).

Angiopoietin-1 induces Tie2 activation, promotes vascular stability, and inhibits vascular permeability [77,78]. Angiopoietin-1 increases cortical actin levels and suppresses endothelial permeability through the activation of Rac1 and suppression of RhoA [36,79]. Moreover, Tie2 activates Rac1 through the activation of IQ domain GTPase-activating protein 1 (IQGAP1), which stabilizes Rac1 GTPase [36] (Figure 5). In addition, Tie2 suppresses RhoA activation through PI3-kinase (PI3K), which activates the RhoA GTPase-activating protein p190RhoGAP [79] (Figure 5). Angiopoietin-1 suppresses the internalization of VE-cadherin induced by permeability-promoting factors and stabilizes VE-cadherin at endothelial cell junctions [80]. 

Angiopoietin-2 is released from the Weibel–Palade bodies of endothelial cells in response to various stimuli and acts both as an agonist and antagonist against of Tie2 [81,82,83]. During inflammation, angiopoietin-2 switches from being an agonist to being an antagonist, increasing vascular leakage. Therefore, as an antagonist, angiopoietin-2 increases vascular permeability via inflammatory cytokines, such as histamine and VEGF [82]. In contrast, angiopoietin-2 decrease vascular permeability in mice [83].

### 3.4. Inflammatory Mediators

Inflammatory mediators, such as histamine, bradykinin, and thrombin, have been shown to induce transient opening of the endothelial barrier. These inflammatory mediators activate receptors coupled with G_q_/G_11_ [84]. G_q/_G_11_ interact with phospholipase C (PLC), which cleaves phosphatidylinositol 4,5-diphosphate (PIP_2_) into inositol 1,4,5-trisphosphate (IP_3_) and diacylglycerol. IP_3_ releases intracellular Ca^2+^ and diacylglycerol, which activates protein kinase C (PKC) [84,85]. It is thought that both intracellular Ca^2+^ release and the transmembrane influx of extracellular Ca^2+^ promote endothelial permeability [86]. However, GPCR-induced endothelial Ca^2+^ signaling is not required for endothelial barrier opening [87].

**Figure 6:**
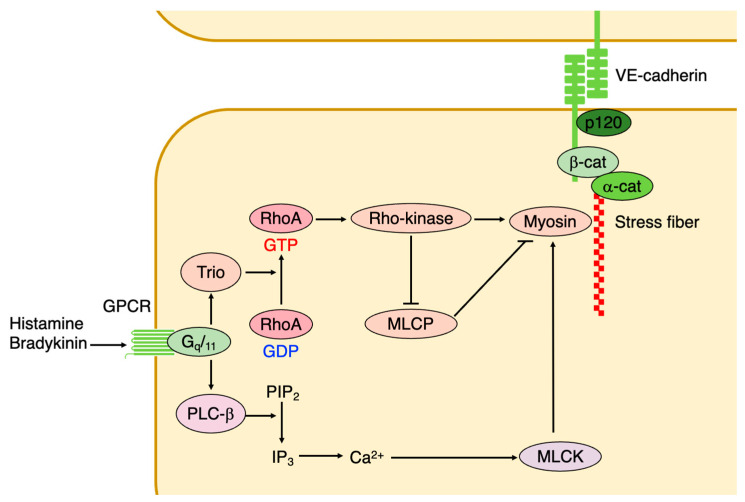
Inflammatory mediators promote endothelial permeability through GPCR. Histamine and bradykinin induce vascular permeability via GPCR. These mediators activate Trio, a RhoGEF, which leads to RhoA activation. Activated RhoA induces actomyosin contraction by activating Rho-kinase and suppressing myosin light chain phosphatase (MLCP).

Thrombin interacts with proteinase-activated receptor (PAR), which is a GPCR. It activates RhoA through the guanine nucleotide exchange factor (GEF), and rearranges cytoskeletal actin in endothelial cells [34,88]. Activated RhoA leads to the activation of Rho-kinase and inhibition of myosin phosphatase, and leads to increases in MLC phosphorylation and actomyosin contraction in endothelial cells [34,89] (Figure 6). Furthermore, RhoA promotes actin stress fiber formation in response to thrombin in endothelial cells and destabilizes vascular integrity [90] (Figure 6). Thrombin activates the non-receptor tyrosine kinase Pyk2, which inhibits between VE-PTP and VE-cadherin interactions, thereby increasing endothelial permeability in a Ca^2+^-dependent manner [91].

Histamine and bradykinin promote endothelial intercellular gap formation and leakage via the VE-cadherin internalization through its phosphorylation at Tyr658 and Tyr685 [19,92]. In addition, histamine promotes actomyosin contraction through G_q_/G_11_, Trio, and RhoA, which alter the localization of the VE-cadherin complex to adherens junctions, forming gaps, and thereby causing vascular leakage [33] (Figure 6). 

### 3.5. Fluid Shear Stress

The lumen of the vascular endothelium is constantly exposed to shear stress related to blood flow. Stable and straight blood flow is observed in straight blood vessels, whereas disturbed flow is present in branching or curved zones. Stable flow leads to the maintenance of the endothelial barrier function, whereas disturbed flow induces endothelial barrier dysfunction [93,94,95,96]. VE-cadherin undergoes tyrosine phosphorylation at Tyr658 and Tyr685 by the Src family of protein tyrosine kinases in response to blood flow [19,97].

Shear stress promotes the expression of the Notch ligand delta-like 4 (Dll4), which activates Notch1 [98,99]. Activated Notch1 is then cleaved, and its transmembrane domain promotes the formation of a complex consisting of VE-cadherin, leukocyte common antigen-related (LAR) tyrosine phosphatase, and Trio [100]. This complex activates Rac1, promoting the assembly of adherens junctions and establishing barrier function [100]. Therefore, shear stress maintains junctional integrity through Notch1, which also mediates intracellular calcium levels in endothelial cells [99]. However, it has also been reported that shear stress-induced Notch1 activation depends on the calcium channel Piezo1, which opens in response to mechanical forces [101]. Some studies have reported that stable blood flow enhances the barrier function of endothelial cells through Tie2 and Kallikrein-related peptidase-10 (KLK10) [102,103]. Stable flow promotes the endocytosis of VE-PTP and its dissociation from Tie2, leading to Tie2 activation [102]. In addition, secreted serine protease KLK10 levels are upregulated by undisturbed blood flow [103]. KLK10 reduces endothelial permeability and reverses the barrier disruption induced by disturbed blood flow [103]. Further, stable blood flow stabilizes tight junctions by upregulating occludin expression and promoting its linkage to the actin cytoskeleton [104].

Disturbed blood flow also activates PAK and the noncatalytic region of tyrosine kinase-1 (Nck1). Activated Nck1 then recruits PAK to the adherent sites and promotes paracellular permeability [105,106]. In addition, G-protein-coupled receptor kinase 2 (GRK2) enhances the disruption of adherens junctions and paracellular permeability through the phosphorylation and inactivation of vinculin in endothelial cells exposed to disturbed blood flow [107]. Disturbed blood flow also promotes LDL transport, which is closely associated with the development of atherosclerosis [93,108,109].

Additionally, disturbed blood flow induces a differentiation transition from endothelial cells to mesenchymal cells (endothelial-to-mesenchymal transition; EndMT), resulting in the loss of intercellular adhesion [110,111]. EndMT is associated with several diseases, including atherosclerosis [111]. 

## 4. Mechanisms of Endothelial Barrier Function in Disease

Endothelial dysfunction and increased permeability play essential roles in many diseases. Under physiological conditions, microvascular permeability is tightly controlled, but a variety of stimuli such as physiological inflammation lead to a transient and brief—therefore, reversible—upregulation of vascular permeability [1,112]. However, permeability control loss leads to various pathological conditions, including hypoxia, chronic inflammatory diseases, tumor angiogenesis, and atherosclerosis [1,112].

In the alveolar capillaries, an abnormal increase in endothelial permeability leads to impaired gas exchange, resulting in hypoxia and death [1]. In addition, elevated pulmonary microvascular inner pressure increases capillary permeability and disrupts barrier function [113]. Under high blood pressure, mice lacking vascular endothelial Piezo1 showed suppressed pulmonary microvasculature edema, leakage, and decreased expression of adherens junction proteins (VE-cadherin, β-catenin, and p120-catenin) [114]. These results suggest that high vascular pressure at the lung endothelial surface is sensed by Piezo1, which dissociates adherens junctions via VE-cadherin endocytosis and degradation, thereby increasing pulmonary microvascular leakage and edema. 

Angiogenesis and microvascular remodeling are features of tissue in chronic inflammatory diseases [115]. Chronic inflammation leads to the formation of leaky capillaries. In tumors, the blood vessels lose their typical hierarchical structure, leading to small leaky arteries, capillaries, and veins [116]. Endothelial dysfunction and increased permeability can also induce atherosclerosis, which is a chronic inflammatory condition characterized by lipid-rich plaque accumulation within the vessel wall [93]. Additionally, in mice with endothelium-specific loss of Piezo1 or Gαq/Gα11, disturbed flow-induced inflammatory signaling and atherosclerosis progression at atherogenic sites are suppressed [117]. Thus, Piezo1 may be involved in disturbed flow-induced inflammatory signaling and atherosclerosis progression. Interestingly, atherosclerotic plaques preferentially develop in the curved and branching regions of arteries, which are associated with disturbed blood flow [118].

Dysfunction of the blood–brain barrier is associated with neurodegenerative diseases, including multiple sclerosis, Alzheimer’s disease, and Parkinson’s disease [119]. Exercise has been suggested to contribute to disease recovery by improving endothelial cell function [120]. In multiple sclerosis, exercise restores the expression of occludin and claudin-4 and maintains the blood–brain barrier in a normal state [121,122]. Aged endothelial cells, in contrast, have low expression of occludin, claudin, and ZO-1, suggesting increased vascular permeability and impaired blood–brain barrier function [123,124].

## 5. Conclusions

The control of vascular endothelial barrier function is essential for tissue homeostasis. Endothelial barrier dysfunction leads to several diseases, such as chronic inflammation, cancer, and atherosclerosis. Therefore, understanding the mechanisms controlling vascular permeability is expected to significantly contribute to the development of preventive and therapeutic strategies against these diseases.
